# Infection due to Buteo buteo herpesvirus in a common buzzard (*Buteo buteo insularum*)

**DOI:** 10.3389/fvets.2023.1152920

**Published:** 2023-05-02

**Authors:** Cristian M. Suárez-Santana, Eva Sierra, Lucía Marrero-Ponce, Ana Colom-Rivero, Jose Navarro-Sarmiento, Simone Segura-Göthlin, Ayoze Castro-Alonso, Óscar Quesada-Canales

**Affiliations:** Unit of Veterinary Histology and Pathology, University Institute of Animal Health and Food Safety (IUSA), Veterinary SchoolUniversity of Las Palmas de Gran Canaria, Las Palmas de Gran Canaria, Canary Islands, Spain

**Keywords:** common buzzard, herpesvirus, Buteo buteo, Canay Islands, raptor pathology, infectious disease, CoHV-1

## Abstract

This study aimed to document the pathological findings observed in a common buzzard (*Buteo buteo insularum*) from Gran Canaria (Canary Islands, Atlantic Ocean), naturally infected with Buteo buteo herpesvirus (HV). Local authorities found the common buzzard alive, but it died after 10 days of specialized veterinary care. Postmortem investigation, including complete gross and histologic examination, immunohistochemistry, microbiology, and PCR, was performed. The animal presented necrotizing heterophilic and histiocytic bilateral conjunctivitis, stomatitis, pharyngitis, rhinitis, and sinusitis with secondary bacterial and fungal infections. Frequent eosinophilic intranuclear inclusion bodies were observed in the oral mucosa and esophagus epithelium. HV proteins and DNA were detected in tissues from this animal. The sequences obtained from the PCR product were identical to the reported sequences of Buteo buteo HV.

## Introduction

An endemic subspecies of the common buzzard (*Buteo buteo insularum*) is present in the Canary Islands (Spain). This raptor population is resident in the islands and strongly territorial (1). Anthropogenic pressure (trauma, electrocution, and poisoning were identified as the significant causes of death) negatively impacts this subspecies (2, 3). While infectious diseases have been suspected in a few cases admitted to the government wildlife hospital of Gran Canaria (2), information about contagious agents in these raptors is limited.

Herpesvirus (HV) infections have been diagnosed in wild birds from America, Europe, the Middle East, Asia, and Australia affecting different avian host species of several different orders, including raptors, columbids, Galliformes, Anseriformes, and psittacines (4–14).

The best-known herpesviral diseases in birds are represented by herpesviruses of Galliformes, including Gallid alphaherpesvirus 1 (GaHV-1) and GaHV-2. GaHV-1 is the causative agent of infectious laryngotracheitis which primarily affects chickens and pheasants, turkeys, partridges, and peafowl. These same hosts have been demonstrated to be susceptible to GaHV-2, which causes Marek’s disease (4, 13). Other herpesviral diseases in wild birds include Pacheco’s disease in psittacines, caused by Psittacid alphaherpesvirus type 1 (PsHV-1), and duck plague in Anseriformes, caused by Anatid alphaherpesvirus type 1 (AnHV-1, also known as duck plague virus or duck virus enteritis) (5). Regarding raptors, the most common herpesvirus reported to cause disease is Columbid alphaherpesvirus type 1 (CoHV-1) (11, 15), responsible for causing inclusion body disease. The same virus causes Smadel’s disease in pigeons (9). Buteo buteo HV infecting common buzzards has recently been identified by molecular methods; however, no descriptions of disease are attributed to this virus in any avian species.

All the abovementioned viral agents belong to the subfamily alphaherpesviridae. The GaHV-1 and PsHV-1 belong to the genus Iltovirus, whereas GaHV-2, CoHV-1, and AnHV-1 are grouped in the genus Mardivirus (4, 16–18). In addition, numerous other herpesviruses are described in the literature, some of which are associated with the disease, while others seem not to produce significant pathology in the host (19–21).

The prevalence of herpesviral infections in buzzards from the Canary Islands is unknown. To the best of the authors’ knowledge, inclusion body disease has not been reported in wild raptors in the archipelago. In this article, we provide a complete case description of herpesviral disease caused by Buteo buteo HV in a common buzzard from Gran Canaria.

## Materials and methods

A common buzzard was found alive in the water of an east coast beach of Gran Canaria in February 2020. Local authorities were informed, and the animal was rescued and immediately transported to the government wildlife hospital of the Cabildo of Gran Canaria, where specialist veterinary assistance was provided. On arrival, the animal was severely dehydrated (5–10%), weighed 710 g, and presented moderate bilateral swelling of the palpebral conjunctiva and third eyelid. Trauma was suspected, and rehydration and administration of antibiotics (intramuscular enrofloxacin and tobramycin collyrium) were performed. Analgesia (oral meloxicam) and a feeding plan were established, but the animal died after 10 days of veterinary care.

The carcass was immediately submitted to the Institute of Animal Health and Food Safety (IUSA), where a comprehensive standardized necropsy was performed on arrival.

### Histology

Standard samples were taken (including adrenal gland, air sacs, bursa of Fabricius, encephalon, esophagus, eyes, heart, intestine, kidney, liver, lung, sciatic nerves, skeletal muscle, skin, spleen, testicles, thymus, thyroid, trachea, and the whole head) and fixed using 4% neutral-buffered formalin for 24 h and routinely processed for histological analysis. In addition, Gram, Periodic Acid Schiff (PAS), Ziehl–Neelsen, Grocott methenamine silver (GMS), Masson’s Trichrome, and von Kossa stains were performed on selected tissue sections as needed.

### Immunohistochemistry

The avidin–biotin complex (ABC) method was used for the immunohistochemical demonstration of the herpesvirus antigen. Formalin-fixed paraffin-embedded (FFPE) tissue sections were mounted on slides precoated with VECTABOND (Vector Laboratories). The sections were deparaffinized and hydrated. Antigen retrieval was performed through heat-induced epitope retrieval with a citrate buffer for 10 min at 95°C. Primary antibody (polyclonal Anti-HSV1 antibody (ab9533) provided by Abcam, Cambridge, UK) was added in a 1/75 dilution and incubated in a humid chamber at 4°C for 18–20 h, followed by blocking of endogenous peroxidase in 0.3% hydrogen peroxide in distilled water for 30 min. The binding between tissue antigens and antibodies was visualized using chromogen 3-amino-9-ethylcarbazole (AEC) for 10 min. A kidney from an HV-positive Blainville’s beaked whale was used as a positive control (22). The same methodology was implemented for the negative control, but the primary antibody was omitted.

### PCR

A maximum tissue input of 25 mg from the air sacs, lungs, kidneys, and esophagus samples of the common buzzard was cut into small pieces before starting DNA extraction through the DNeasyTM Blood & Tissue Kits (Qiagen, Inc., Valencia, CA, United States). Additionally, genomic extraction from a fibrinonecrotic exudate swab collected from the conjunctiva of the individual was also performed using the same kit. Samples were placed in a 1.5 ml microcentrifuge tube in which 20-μL proteinase K was subsequently added with an occasional vortex at 56°C during 15 min of incubation. When complete lysis of the tissues was achieved, the DNA extraction was continued according to the manufacturer’s instructions without any adaptations. Finally, 50 μl of DNA-eluted samples were obtained. A pan herpesvirus conventional nested polymerase chain reaction (PCR) was performed for HV detection. A fragment of the DNA polymerase gene of the Herpesviridae family of about 200 bp was amplified according to a previously published protocol (23) adapted to our laboratory conditions. Specifically, DNA templates (2 μL) were amplified in a reaction mixture containing 2.5 mM of each buffer (10× and MgCl_2_), 0.4 μM of each PCR primer, 0.2-mM deoxynucleotide triphosphate, and 0.05 U/μL of Taq DNA polymerase (Roche Applied Science) and diethylpyrocarbonate (DEPC)-treated water to a total reaction volume of 12.5 μL. Primary and secondary PCRs were performed under the same conditions: the initial denaturation step was performed at 94°C for 5 min, followed by 45 cycles with 30 s denaturation at 94°C, 60 s of annealing at 46°C, and 60 s of strand extension at 72°C. After cycling, the reaction mixtures were incubated for 7 min at 72°C. The negative and positive controls were included. Horizontal gel electrophoresis was performed in 2% agarose containing GelRed® (Biotium, Inc., CA, United States) for 5 μL of the obtained amplicons from the second PCR. Purification of PCR products was carried out using a Real Clean Spin kit (REAL®, Durviz, S.L., Valencia, Spain) to perform bidirectional sequencing (the Sanger method) (Secugen S.L., Madrid, Spain) with 1 μL (5 μM) of TGV (internal forward) and IYG (internal reverse) primers. The amplicon identities were confirmed with BLAST.^1^ The HV sequences obtained (excluding primers) were aligned through the ClustalW algorithm using MEGAX software (Pennsylvania, PA, United States) (24, 25) to attain a consensus sequence.

### Phylogenetic analysis

A phylogenetic tree was constructed using the maximum likelihood statistical method with Tamura’s 3-parameter model (T92 + G + I) for nucleotides (26). Bootstrap resampling (500 replicates) was used to assess the reliability of the tree. Suid betaherpesvirus (GenBank AF268040) was used as an outgroup in the tree.

### Microbiology

Microbiological analysis was performed with frozen samples obtained from the esophagus, kidney, lung, and infraorbital caseum. Samples were cultured on routine agars and incubated at 37°C for 24 h under aerobic and anaerobic conditions (4–10% CO_2_ using a BBL GasPak Plus system).

## Results

### Gross pathology

**Figure 1:**
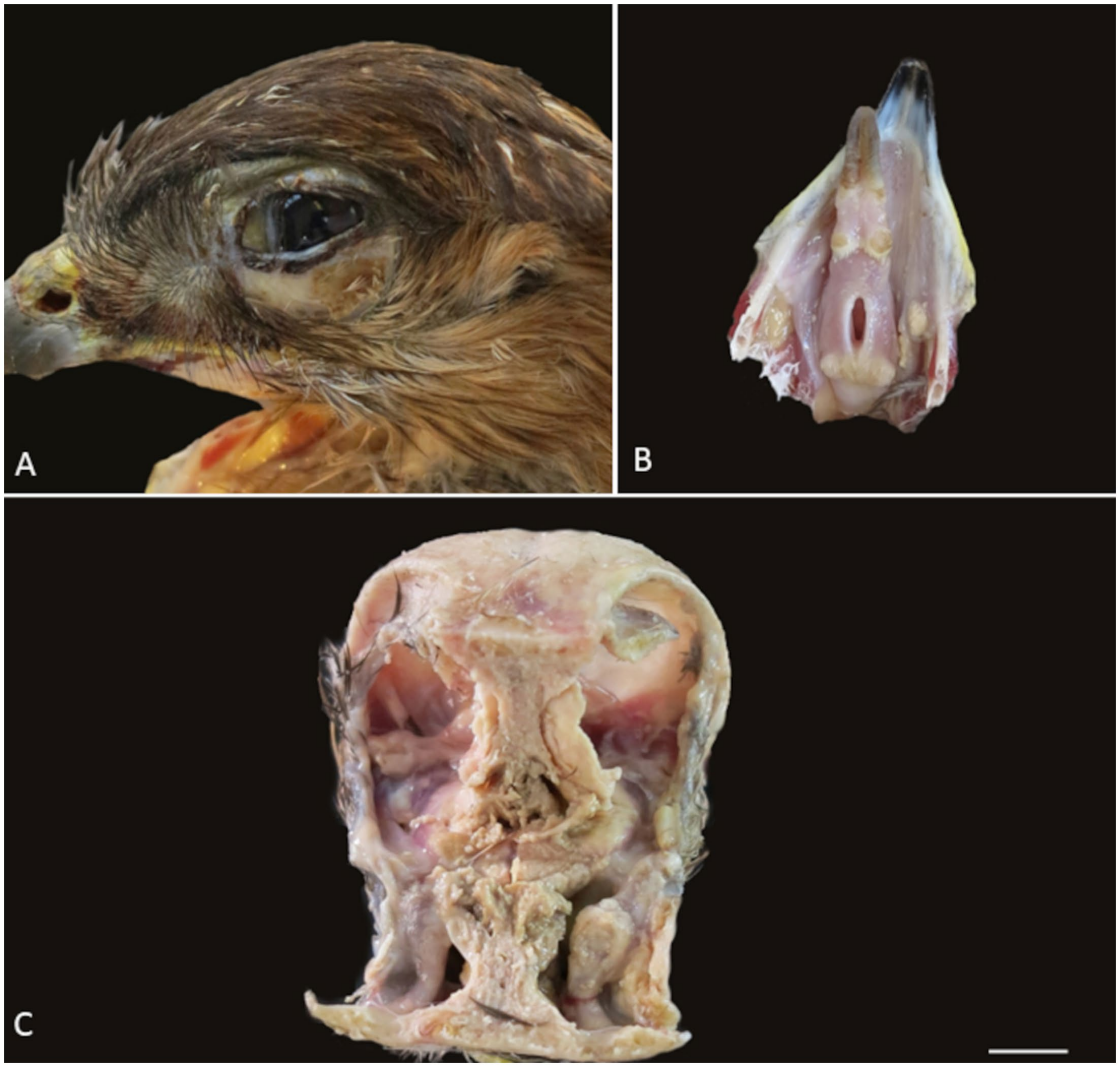
Gross lesions observed during the necropsy of the common buzzard. **(A)** Periorbital alopecia with fibrinous exudate and swelling of the conjunctiva. **(B)** Multifocal fibrinonecrotic plaques on the tongue and oral mucosa. **(C)** Nasal chambers and infraorbital sinus filled with abundant friable yellow exudate (caseum).

The animal was in poor body condition, weighing 614 g, with moderate atrophy of the pectoral muscles and serious atrophy of the visceral and epicardial fat. Bilateral periorbital alopecia, diffuse swelling of the conjunctiva and multifocal yellow areas on the skin of the eyelids were noted (Figure 1A). Multifocal yellow plaques were observed in the oral cavity, including on the tongue, hard palate, and pharynx (Figure 1B). Infraorbital sinuses and nasal cavities were filled by abundant friable yellow exudate (caseum) that infiltrated the nasal conchae bilaterally and extended to the infraorbital sinuses (Figure 1C). The liver was diffusely pale, and the air sacs were slightly opaque.

### Histopathology

**Figure 2:**
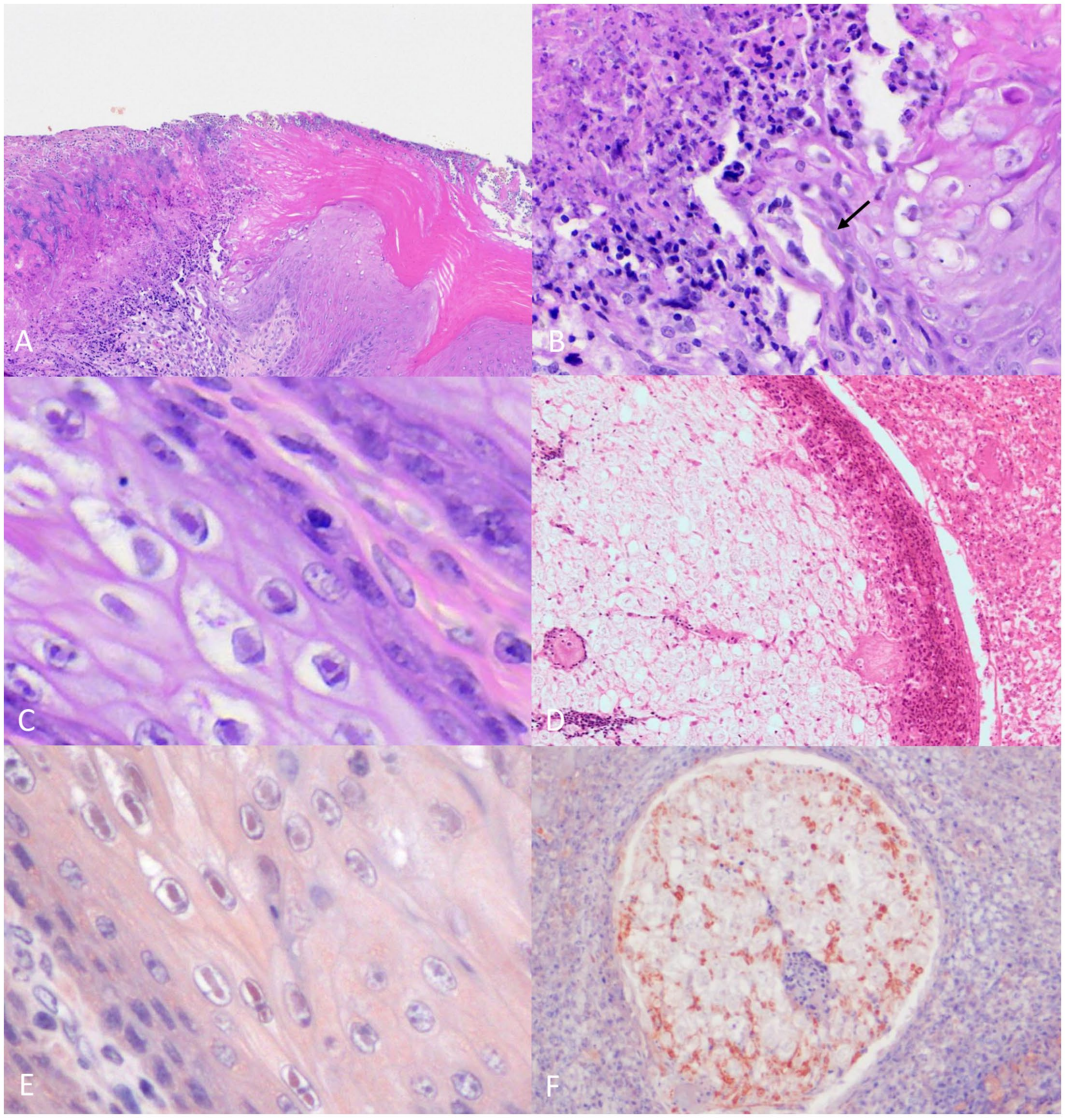
Histopathological findings of the common buzzard. **(A)** Necrosis of the oral mucosa covered by abundant fibrinoheterophilic exudate. 10× H&E stain. **(B)** Higher magnification of the same area of panel **A**. Detail of the mucosal necrosis and presence of intralesional mycotic hyphae (arrow). 40× H&E stain. **(C)** Tongue epithelium showing eosinophilic intranuclear viral inclusion bodies. 60x H&E stain. **(D)** Optic branch of the trigeminal nerve with neuritis and perineuritis (10×). **(E)** Esophageal epithelium showing intranuclear positive immunolabeling for HSV. 60× IHC. **(F)** Trigeminal nerve branch with intra-axonal immunolabeling for HSV. 20× IHC.

The oral cavity (including the tongue and nasopharynx), the nasal cavity, and the conjunctiva show multifocal, moderate-to-severe mucosal necrosis, as shown in Figures 2A,B, and ulceration with vacuolar degeneration of epithelial cells that frequently contained round 2–4-μm diameter eosinophilic intranuclear viral inclusion bodies surrounded by a clear halo (Cowdry type A inclusions) is shown in Figure 2C. Similar but less severe lesions and occasional Cowdry Type A inclusion bodies were observed multifocally in the esophageal mucosa.

In addition, multifocal, extensive areas of necrosis and abundant histiocytic and heterophilic infiltration invaded the underlying oral and pharyngeal stroma, with occasional multinucleated giant cells. The presence of scattered multifocal bacterial colonies (cocci) and oval—3-μm diameter—GMS and PAS-positive individual yeasts (most likely *Candida* sp.) frequently arranged in short chains (pseudohyphae) and hyphae on the mucosal surface are shown in Figure 2B.

There were multiple blood vessels in the nasal cavity with thrombosis, subintimal fibrin deposition, necrosis, and histiocytic inflammation of the tunica media (vasculitis). The lumen of the affected vessels was entirely occluded by fibrin thrombus admixed with leukocytes and a few erythrocytes and, occasionally recanalized. The maxillary and optic branches of the trigeminal nerve showed periaxonal vacuolization, satellitosis, necrosis and neuronophagia of ganglion neurons, and infiltration of lymphocytes. Additionally, the perineum was markedly infiltrated by histiocytes and degenerated heterophils as shown in Figure 2D.

The eyelids presented moderate to severe epidermal necrosis and ulceration with large heterophilic granulomas admixed with coccoid bacteria. Extensive areas of necrosis and infiltration of heterophils, macrophages, and multinucleated giant cells expanded, infiltrated, and replaced the nasal cornetts, nasal septum, lacrimal glands, and infraorbital sinuses. In contrast, the nasal passages were filled by necrotic debris and large numbers of degenerated and viable heterophils, with scattered coccoid bacteria. No fungal structures were observed in the nasal passages or sinuses.

Other histopathological findings include mild multifocal necrosis of hepatocytes, moderate lymphohistiocytic air sacculitis, moderate hepatosplenic hemosiderosis, the presence of multiple nematodes in the tongue epithelia (most likely *Capillaria* sp.) with no inflammatory reaction, and focally extensive, severe myocyte necrosis in the pectoral muscle.

### Virus identification and phylogenetic analysis

The inclusion bodies observed in the tongue, oral cavity, and esophagus keratinocytes, showed immunolabeling for herpes simplex virus (HSV) (Figure 2E). In addition, the different branches of the trigeminal nerve showing strong axonal immunolabeling for HSV antibodies are shown in Figure 2F.

**Figure 3:**
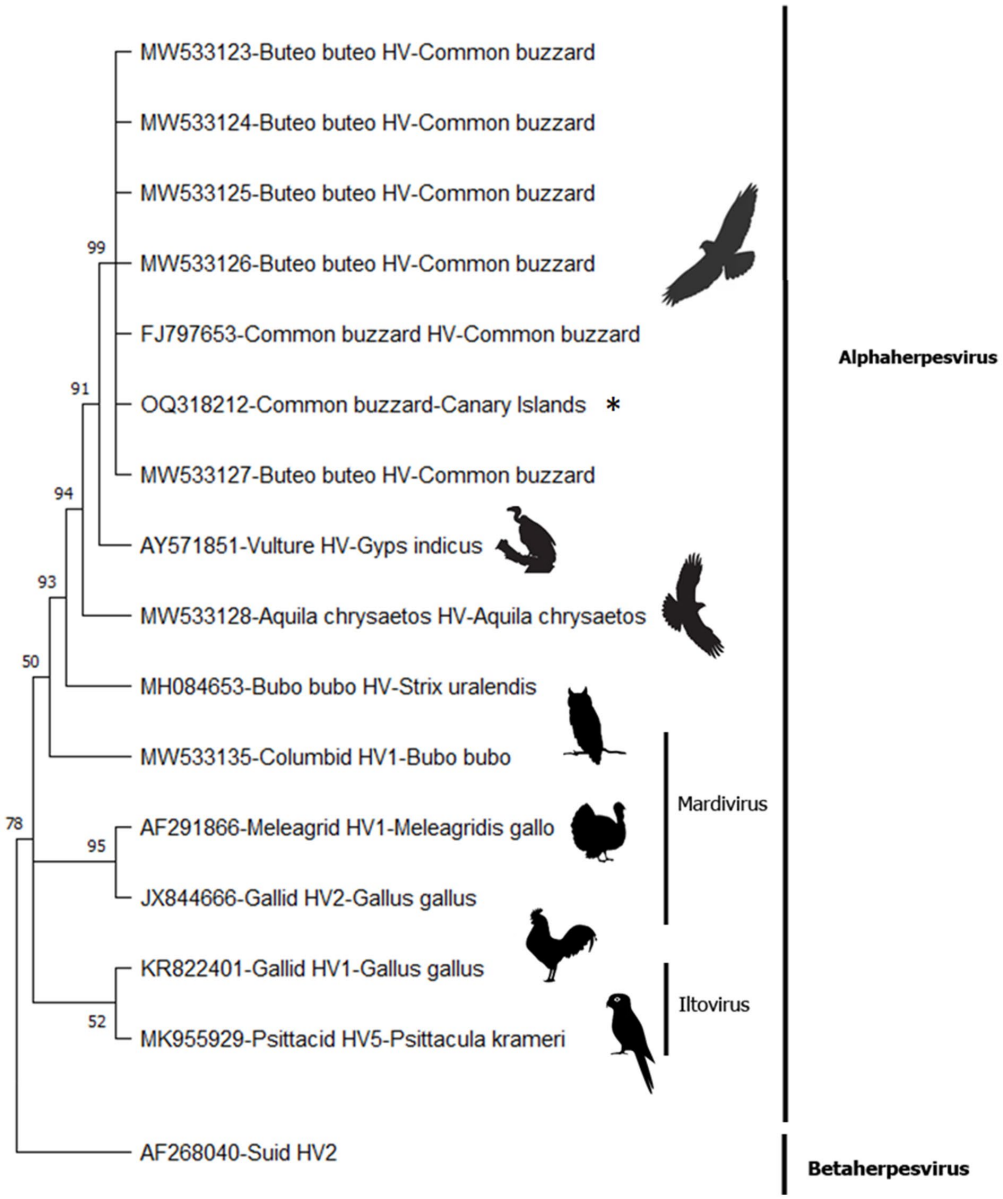
Condensed phylogenetic tree (50% bootstrap cutoff value) constructed from DNA polymerase partial sequences of herpesvirus species using the maximum likelihood method with Tamura’s 3-parameter model (T92 + G + I) for nucleotides. The asterisk denotes the fragment amplified from the common buzzard from Gran Canaria.

Molecular methods detected the presence of herpesvirus DNA in the esophagus, air sacs, lung, and kidney samples. All the obtained sequences were identical. The consensus sequence was 181 base pairs in length (excluding primers) and corresponded (100% nucleotide identity with 100% query cover) with previously published Buteo buteo HV (Figure 3). Three main branches were created within the avian alphaherpesviruses, including sequences belonging to *Iltovirus* (52% bootstrap value [BV]), *Mardivirus* (95% BV), and some unclassified viruses detected in raptor species (50% BV). Within the raptor group, all the previously published sequences, identified as Buteo buteo HV, were clustered with a 99% BV and were in the same clade as a sequence detected in an Indian vulture (*Gyps indicus*) (91% BV). The possibility of a common ancestor of these sequences—and a sequence seen in a golden eagle (*Aquila chrysaetos*)—is supported by a 94% BV. Strong support (93% BV) for a common ancestor of these herpesvirus sequences was also detected in different species within the order Accipitriformes and the *Bubo bubo* HV (order Strigiformes).

### Microbiology

Pure growth of *Enterococcus faecalis* was obtained from the lung, kidney, and esophagus samples. No bacterial or fungi growth was detected in samples from the periorbital caseous material.

## Discussion

This common buzzard presented severe necrotizing stomatitis, pharyngitis, rhinitis, and sinusitis associated with intralesional Cowdry Type A inclusions, and herpesvirus infection, which was confirmed by the immunohistochemistry (IHC) and PCR results. Buteo buteo HV was identified following the sequencing of the PCR product.

Different herpesviruses are known to cause fatal diseases in chickens, including Marek’s disease (Gallid HV-2) and infectious laryngotracheitis (Gallid HV-1), which are globally widespread. Although Gallid HV-2 occurs with neoplasia, Gallid HV-1 frequently occurs with conjunctivitis, rhinitis, and infraorbital sinusitis (27), as observed in this case. However, clinicopathological presentations of herpesvirus diseases may differ in wild birds, ranging from respiratory problems, conjunctivitis, diarrhea, anorexia, regurgitation, and biliverdinuria to sudden death (28).

CoHV-1 (previously pigeon herpesvirus, falcon Herpesvirus, and stringid herpesvirus) was first described from domestic pigeon (*Columba livia*) lofts in the United States in 1945 (29) and has subsequently been documented in pigeons around the world (6, 8, 14). CoHV-1 causes a multisystemic disease with high mortality (4, 7) when introduced into a naïve or immunosuppressed population of pigeons. Birds that survive are subclinically infected for life and shed the virus during the breeding season. Their young are protected from disease by the transfer of maternal antibodies but can still become latently infected (30). An initial viral infection that occurs in the mucosa of the conjunctiva, respiratory, and digestive tracts is followed by viremia. Conjunctivitis and fibrinonecrotic exudate in the nasopharynx are common findings (4).

CoHV-1 is also recognized as the etiologic agent of inclusion body disease or inclusion body hepatitis in birds of prey (8, 9, 31, 32). Inclusion body disease is characterized by hepatic, splenic, and bone-marrow necrosis. The virus can infect different species of falcons, owls, and columbiformes (32); though some species of raptors seem resistant to the disease (7). Experimental infections indicate that the virus can cross between different avian species, including non-raptorial (33). Natural infections of raptors with CoHV-1 have been observed in North America, Eurasia, and Australia (8, 9, 11, 14). In contrast to the natural infection of CoHV-1 in columbiformes, which usually causes low mortality because of transmission of maternal immunity, the infection of CoHV-1 in raptors usually causes mortality rates approaching 100% (8, 33). Infection with CoHV-1 in raptors has been associated with the consumption of infected pigeons (4, 11).

A previous report described lesions resembling inclusion body disease in one common buzzard from Spain, confirmed to be naturally infected by a herpesvirus (34). In the Canary Islands, there are previous reports of an outbreak of encephalitis caused by a herpesvirus in domestic doves (*C. livia*) (35). However, in both cases, the viruses were identified using electron microscopy, and molecular analysis was not undertaken to better characterize the herpesviral infections. These diseases could have been produced by either CoHV-1 or other herpesviruses (for example, Buteo buteo HV), however, the epidemiological and pathological presentations suggest CoHV-1.

The lesions in this buzzard are similar to those of pigeons infected with CoHV-1 and chickens infected with Gallid HV-1, in which necrotic and fibrinoheterophilic lesions predominate in the conjunctiva, oral mucosa, and epithelium of the nasopharynx, with coinfections of opportunistic pathogens. The pathogenesis of Buteo buteo HV infection in the common buzzard may be more comparable with that described in herpesviruses adapted to their host and not the systemic presentation noted in non-definitive hosts, such as raptors infected with CoHV-1. The histopathology revealed neuritis and perineuritis of the maxillary and optic branches of the trigeminal nerve and showed axonal IHC-labeling for Herpesvirus. It can be expected that Buteo buteo HV acts similarly to other alphaherpesviruses with persistent infection in sensory ganglia or mononuclear blood cells. Stress, trauma, or other immunosuppressive factors would trigger reactivation and direct neural spread to the nasal cavity, conjunctiva, and oral cavity through the maxillary and mandibular branches of the trigeminal nerve (17).

According to our phylogenetic tree, the closest related viruses to Buteo buteo HV would be a herpesvirus (AY571851) detected in an Indian vulture (36), and another herpesvirus (MW533128) identified in a golden eagle (14), both within the same order and family (order Accipitriformes; family Accipitridae) as the common buzzard. The phylogenetic tree also shows that Buteo buteo HV is genetically closely related to other HVs detected in the order Strigiformes, family Strigidae, but more differentiated from other avian herpesviruses such as CoHV-1, Gallid HV-1, and PsHV-1. Until now, Buteo buteo HV has only been detected in the common buzzard; however, if this herpesvirus acts similarly to other avian alphaherpesviruses, cross-infection between different species may be possible if the circumstances are favorable (i.e., different avian species coexisting in an enclosed area).

Although a previous report described an outbreak of encephalitis caused by a herpesvirus affecting domestic doves in the Canary Islands (35), potentially caused by CoHV-1, there are no epidemiological studies of the presence of herpesvirus in birds from this geographical area. Little is known about how herpesviruses can circulate and adapt across different avian species and how these agents may affect endemic bird populations.

In our case, swelling inflammation of the palpebral conjunctiva and third eyelid was noted on arrival at the hospital and suggested that the animal was already affected by the virus before medical treatment. It is possible that conjunctivitis impaired the animal’s ability to hunt and predisposed it to disorientation and nutritional imbalance. Pectoral muscle necrosis was observed in the injection sites of intramuscular drugs. Although the animal was treated with antibiotics, numerous bacteria were observed in the necrotic debris of the nasal cavity and infraorbital sinuses. Additionally, the manifestation of fungal coinfection complicates the clinical presentation of the case. Correct diagnosis upon arrival of the animal at the hospital and application of antiviral medication may help the recovery of other individuals with this presentation. Recognition of compatible lesions and confirmation of the infection from PCR of oropharyngeal swabs may be the best diagnostic approach in live animals. However, further studies are necessary to understand the prevalence of the virus in buzzard populations.

Our results indicate that Buteo buteo HV can cause fatal diseases in common buzzards characterized by conjunctivitis, rhinitis, and stomatitis. This is the first report of disease caused by Buteo buteo HV in a common buzzard.

## Data availability statement

The datasets presented in this study can be found in online repositories. The names of the repository/repositories and accession number(s) can be found below: https://www.ncbi.nlm.nih.gov/genbank/, BankIt2666284 Seq1 OQ318212.

## Ethics statement

Ethical review and approval was not required for the study on death animals in accordance with the local legislation and institutional requirements.

## Author contributions

CS**-**S: writting of the manuscript, Necropsy, histology, and immunohistochemistry. EV: molecular characterization of the virus and histological diagnosis. LM-P: histochemistry and histopathology. AC-R: molecular characterization of the virus. JN-S: histochemistry and histopathology. SS-G: immunohistochemistry and molecular characterization of the virus. AC**-**A: histochemistry and histopathology. OQ-C: necropsy, histology, histochemistry, and immunohistochemistry. All authors contributed to the article and approved the submitted version.

## Funding

This study has been performed with economical and logistical support from the “Dirección General de Lucha Contra el Cambio Climático y Medio Ambiente” under the creation of the Canarian Network for the Surveillance of the Wildlife Health (Orden *N*°134/2020 de 26 de mayo de 2020). Publication fees have been charged by the project ULPGC Excellence, funded by the Consejería de Economía, Conocimiento y Empleo del Gobierno de Canarias.

## Conflict of interest

The authors declare that the research was conducted in the absence of any commercial or financial relationships that could be construed as a potential conflict of interest.

## Publisher’s note

All claims expressed in this article are solely those of the authors and do not necessarily represent those of their affiliated organizations, or those of the publisher, the editors and the reviewers. Any product that may be evaluated in this article, or claim that may be made by its manufacturer, is not guaranteed or endorsed by the publisher.

## Footnotes

^1^ www.ncbi.nlm.nih.gov/blast/Blast.cgi
